# Special Issue—“Bioactive Compounds from Natural Sources II”

**DOI:** 10.3390/molecules28114450

**Published:** 2023-05-31

**Authors:** Oksana Sytar, Iryna Smetanska

**Affiliations:** 1 Institute of Plant and Environmental Sciences, Slovak Agricultural University in Nitra, 94976 Nitra, Slovakia; 2 Department of Plant Food Processing, Agricultural Faculty, University of Applied Sciences Weihensteph-an-Triesdorf, Markgrafenstr 16, 91746 Weidenbach, Germany; iryna.smetanska@hswt.de

In recent decades, there has been a great interest in bioactive compounds from natural sources. Plant biodiversity proposes a large number of bioactive compounds. Bioactive compounds are mostly some specific secondary metabolites with antioxidant, inflammatory, immunomodulating potential, antimicrobial properties, etc. The bioactive compounds of classes of terpenes, flavonoids, alkaloids, coumarins, stilbenes, etc., alongside the description of some of their mechanisms of action, are important to study. It is important to admit that the use of plant extracts complicates the identification of the effects of antiviral, antimicrobial, or other capacities of separate biologically active compounds, and the effects of activities of solute compounds in extracts can be additive (the synergistic effect), or an antagonistic effect can come into play. In that context, the current issue is open for scientific research on the description of novel isolated bio-active compounds, as well as some of their unknown effects, which are a priority for the applied needs of humans in different areas of life. Due to a strong interest in the current topic in the previous Special Issue, we continued to collect unique and interesting research works connected with plant natural compounds and their biological effects.

Four review papers are in the current Special Issue. A comprehensive review of recent advances in chiral A-ring flavonoid-containing compounds (structure, bioactivities, and synthesis described the research progress of chiral A-ring-containing flavonoids, including isolation and extraction, structural identification, pharmacological activities, and synthetic methods, which were comprehensively and systematically summarized. Furthermore, it provided suggestions for future research on the synthesis and biomedical applications of flavonoids [1]. The specific role of flavonoids has been discussed in the review article “Molecular shield for protection of buckwheat plants from UV-B radiation” [2]. The role of rutin and phenolic acids from Thevetia peruviana Latex, with molecular docking of antimicrobial and anticancer activities, was studied [3]. At the same time it was found that rutin promotes pancreatic cancer cell apoptosis by upregulating miRNA-877-3p expression [4].

A comprehensive analysis of the chemical structures and bioactivities of most representative specialized metabolites isolated from the medicinal herb Teucrium species (family Lamiaceae) was performed [5]. The article described representatives of iridoids and iridoid glycosides isolated from Teucrium chamaedrys, Teucrium oliverianum, and Teucrium polium. Representative neo-clerodane diterpenoids isolated from Teucrium chamaedrys and Teucrium scordium subspecies of scordioides were presented. Representative neo-clerodane diterpenoids, sesquiterpenoids, abeo-abietane diterpenoids, triterpenoids, and sterols isolated from Teucrium polium were found as well. The bioactivity and pharmacological properties of identified compounds are shown. 

A review article on the mechanistic insight of plant-derived anticancer bioactive plant compounds and their structure and activities relationship discusses the role of ellipticine, camptothecin, combretastatin, curcumin, homoharringtonine, and other plant-derived bioactive plant compounds with potential anticancer properties. The role of new methods in advanced analytical chemistry and computational tools of analysis has been presented. At the same time, the investigation of new strategies for administration, such as nanotechnology, may enable the development of plant compounds as drug products [6].

Four novel indane derivatives, anisotindans A–D, were isolated from the roots of Anisodus tanguticus. Their structures were known using comprehensive spectroscopic analyses, and their absolute configurations were determined by electronic circular dichroism (ECD) calculations and single-crystal X-ray diffraction analyses. All characteristics and parameters were described [7]. Liu et al., 2022 isolated seven new coumarinolignans, walthindicins A–F, along with five known analogs from the roots of Waltheria indica [8]. It is important to describe novel secondary metabolites in the various medicinal herbs and crop plants due to their further applied use. Plant biodiversity may have a significant impact on the development of new plant-based drugs or original nutritional foods. It studied the anatomical and phytochemical characteristics of different parts of Hypericum scabrum L. extracts, essential oils, and their antimicrobial potential. The GC-FID and GC-MS analyses showed that major components of roots, aerial parts, flowers, and fruit oils were undecane (66.1%), α-pinene (17.5%), γ-terpinene (17.4%), and α-thujene (16.9%), as well as α-pinene (55.6%), α-thujene (10.9%), and γ-terpinene (7.7%), and. Finally, α-pinene (85.2%), respectively [9].

A novel bromoindole alkaloid Geobarrettin D was isolated from the marine sponge Geodia barretti, which was collected from Icelandic waters [10]. It was found that Geobarrettin D may incur anti-inflammatory activity via decreasing secretion of the pro-inflammatory cytokine IL-12p40 by human monocyte derived dendritic cells, without affecting the secretion of the anti-inflammatory cytokine IL-10 [10].

Novel phytochemicals with anticholinesterase activity were estimated in the extracts of Cladonia portentosa. Cladonia portentosa is commonly known as reindeer lichen found in the Irish boglands. To identify the active components, the extract was deconvoluted using a successive extraction process with hexane, ethyl acetate, and methanol to isolate the active fraction. Olivetolic acid, 4-O-methylolivetolcarboxylic acid, perlatolic acid, and usnic acid were isolated and analyzed using ESI-MS and two-dimensional NMR techniques. The LC-MS technique also determined the evidence of the additional usnic acid derivatives, as well as placodiolic and pseudoplacodiolic acids. Analysis of the isolated components confirmed their anticholinesterase potential [11].

A commercially available hit compound, chicoric acid, was evaluated for its anti-cancer potential against PHGDH-expressing gastric cancer cell lines (MGC-803 and SGC-7901), as well as cell lines with low expression of PHGDH (MCF-7 and MDA-MB2-31), which demonstrated that chicoric acid possesses selective cytotoxicity toward PHGDH expressing cancer cell lines [12]. The potential of phenolic compounds was unveiled, which could serve as novel candidates for the development of human 3-phosphoglycerate dehydrogenase (PHGDH) inhibitors as anti-cancer agents [12].

HPLC flavonoids from Ginkgo biloba leaf extracts were detected as apigenin, luteolin, myricetin, and catechin, while HPLC phenolic compounds were pyrogallol, caffeic acid, gallic acid, and ellagic acid. The present study aimed to estimate the antiviral activities of Ginkgo biloba leaf extracts and eco-friendly free silver nanoparticles (Ag NPs) against the MERS-CoV (Middle East respiratory syndrome-coronavirus) and HCoV-229E (human coronavirus 229E), as well as isolation and identification of phytochemicals from Ginkgo biloba [13].

Together with medicinal plants, this presented Special Issue is presented to study and assess the nutritional quality of soybeans concerning fatty acid profile and pesticide residue contamination [14]. Assessing the quality of Burkina Faso soybeans, based on fatty acid composition and pesticide residue contamination, showed a significant variation in the quantitative and qualitative fatty acid composition of the two soybean varieties. The profile of fatty acids and the content of pesticide residues were used as important determinants for soybean utilization in human nutrition [14].

Effects of sonication and thermal pasteurization on the nutritional, antioxidant, and microbial properties of noni juice have been studied. Sonication is recognized as a potential food processing method to improve the functional properties of fruit juice. This study evaluated the effects of different sonication durations (20, 40, and 60 min) and thermal pasteurization on the nutritional, antioxidant, and microbial properties of noni juice. Fresh noni juice served as the control. The main organic acids detected were malic and ascorbic acids [15].

Therefore, this presented Special Issue is a significant contribution to the area of plant natural compounds, which covers research studies from different parts of the world to highlight the main research topics in the research of plant secondary metabolites for human needs.
